# Extracellular polysaccharides from Ascomycota and Basidiomycota: production conditions, biochemical characteristics, and biological properties

**DOI:** 10.1007/s11274-015-1937-8

**Published:** 2015-09-04

**Authors:** Monika Osińska-Jaroszuk, Anna Jarosz-Wilkołazka, Jolanta Jaroszuk-Ściseł, Katarzyna Szałapata, Artur Nowak, Magdalena Jaszek, Ewa Ozimek, Małgorzata Majewska

**Affiliations:** Department of Biochemistry, Institute of Biology and Biochemistry, Maria Curie-Skłodowska University, Akademicka 19, 20-033 Lublin, Poland; Department of Environmental Microbiology, Institute of Microbiology and Biotechnology, Maria Curie-Skłodowska University, Akademicka 19, 20-033 Lublin, Poland

**Keywords:** Exopolysaccharides, Fungi, Ascomycota, Basidiomycota, Biological properties

## Abstract

Fungal polysaccharides (PSs) are the subject of research in many fields of science and industry. Many properties of PSs have already been confirmed and the list of postulated functions continues to grow. Fungal PSs are classified into different groups according to systematic affinity, structure (linear and branched), sugar composition (homo- and heteropolysaccharides), type of bonds between the monomers (β-(1 → 3), β-(1 → 6), and α-(1 → 3)) and their location in the cell (cell wall PSs, exoPSs, and endoPSs). Exopolysaccharides (EPSs) are most frequently studied fungal PSs but their definition, classification, and origin are still not clear and should be explained. Ascomycota and Basidiomycota fungi producing EPS have different ecological positions (saprotrophic and endophytic, pathogenic or symbiotic-mycorrhizae fungi); therefore, EPSs play different biological functions, for example in the protection against environmental stress factors and in interactions with other organisms. EPSs obtained from Ascomycota and Basidiomycota fungal cultures are known for their antioxidant, immunostimulating, antitumor, and antimicrobial properties. The major objective of the presented review article was to provide a detailed description of the state-of-the-art knowledge of the effectiveness of EPS production by filamentous and yeast Ascomycota and Basidiomycota fungi and techniques of derivation of EPSs, their biochemical characteristics, and biological properties allowing comprehensive analysis as well as indication of similarities and differences between these fungal groups. Understanding the role of EPSs in a variety of processes and their application in food or pharmaceutical industries requires improvement of the techniques of their derivation, purification, and characterization. The detailed analyses of data concerning the derivation and application of Ascomycota and Basidiomycota EPSs can facilitate development and trace the direction of application of these EPSs in different branches of industry, agriculture, and medicine.

## Introduction

Microbiological polysaccharides (PSs) are classified into different groups according to their location in the cell, structure, sugar composition, type of bonds between monomers, and systematic affinity. Exopolysaccharides (EPSs) are the most frequently studied microbial PSs, besides cell wall PSs and intracellular cytosolic PSs. Several definitions of EPS are reported in the literature (Mahapatra and Banerjee 2013b). In many cases, EPSs are rather defined by the separation/extraction method used than by theoretical consideration of the composition of the cell wall and macromolecules outside the cell wall. Bound or soluble EPSs contain not only high-molecular-weight polymeric compound exudates from microorganisms, but also products of cellular lysis and hydrolysis of macromolecules.

EPSs are particularly intensively studied in bacterial cultures, where they are synthesized in high concentrations in young cultures growing on various carbon (C) and nitrogen (N) sources, but at a relatively high temperature (Donot et al. 2012). Filamentous and yeast Ascomycota and Basidiomycota fungi are used for biotechnological derivation of EPSs in laboratory conditions using similar techniques, culture conditions, sources of C, N and other elements, culture periods, and mostly acidic pH of the medium (Shu and Lung 2004; Mahapatra and Banerjee 2013b). Fungal strains belonging to both these groups are an important component, characterized by the highest biomass, of the microbiome of various environments (Olsson et al. 1999). It worth mentioning that the optimal period of EPS synthesis by these fungi does not correspond to the period of biomass formation (Madla et al. 2005; Shih et al. 2008). Ascomycota and Basidiomycota fungi exhibit a very high variation of the weight of synthesized EPSs and their biochemical and biological properties (Donot et al. 2012; Mahapatra and Banerjee 2013b).

Microbial PSs are the subject of research in many fields of science. Many properties of PSs have already been confirmed and the list of postulated functions continues to grow. The directions of research on PSs are mainly focused on identification of factors responsible for their synthesis/release, optimization of production (connected with the cost and productivity), and elucidation of the role and application of PSs in interactions between various organisms. The knowledge of the diversity of fungal PSs and the mode of interaction between PSs with microorganisms and higher organisms is still limited, since identification of PSs and investigation of the mechanisms of this interaction depends on the availability of isolation and diagnostics methods.

PS isolation methods are based on fractionation and use of various solvents and PS procedures. Lack of uniformity in these methods often makes the results of different studies incomparable, or even contradictory. Understanding the role of PSs in a variety of processes and the application of PSs in food or pharmaceutical industries or as plant resistance inducers (elicitors) requires improvement of the techniques of derivation, purification, and exploration of properties. The process of PS derivation involves selection of suitable microorganisms, the type of culture, and the method for PS preparation/extraction. Fungal EPSs are derived through ethanol precipitation with different proportions of the culture/water suspension and alcohol (Shu and Lung 2004; Chen et al. 2011; Ma et al. 2015).

Exploration of the similarities and differences in the conditions as well as the efficiency of EPS production and derivation is of great importance as it could contribute to standardization of technologies and selection of optimum production conditions and techniques of purification of fungal EPSs. Very important is also identification of the similarities and differences in the structure and properties of EPSs produced by Ascomycota and Basidiomycota strains, which could facilitate development of mixed formulas containing both Ascomycota and Basidiomycota mycelia or the EPSs of these strains. Given the complementarity of their components, such preparations should have a broader spectrum of activity in various fields of biotechnology, medicine, agriculture, and environmental protection.

## Production of EPS by Ascomycota and Basidiomycota fungal strains

### Parameters of the growth medium and culture conditions

Recently, many fungal filamentous and yeast Ascomycota and Basidiomycota strains, are known for their ability to produce EPSs in various culture conditions. The most commonly encountered EPS producers from these both group of microorganisms and parameters of culture growth facilitating EPS production are collected, selected, and presented in Table 1.

**Table 1:** Ascomycota and Basidiomycota strains producing EPS: the composition of growth medium and the culture conditions

EPS producer	Main parameters of growth medium, type of medium and concentration of components (g l^−1^)			Culture conditions				EPS yield (g l^−1^)	References
	Carbon source	Nitrogen source	Salts	Type of culture (rev. min^−1^)	Temperature (°C)	Initial pH	Optimal time (day)		
Ascomycota									
*Alternaria alternate*	Glucose (40)	YE (20)	KH_2_PO_4_ (0.5), MgSO_4_·7H_2_O (0.5)	Shaking (150)	30	6.58	9	11.96	Nehad and El-Shamy (2010)
*Aspergillus* sp. Y16	Glucose (20)	YE (3), Peptone (5), ME (3)	KH_2_PO_4_ (0.5), NH_4_Cl (0.5), Sea salt (24.4)	Shaking (–)	25	6.0–6.5	7	0.60	Chen et al. (2011)
*Aspergillus* sp. RYLF17	Glucose (–)	PE in PDB	–	Fermentation	30	6.0	14	0.60	Yadava et al. (2012)
*Aspergillus versicolor*	Sorbitol (20), maltose (20)	MG (10), YE (3), tryptophane (0.5)	KH_2_PO_4_ (0.5), MgSO_4_·7H_2_O (0.3), sea salt (33.3)	–	20	6.5	30	0.24	Chen et al. (2013a)
*Aureobasidium pullulans* RYLF-10	Sucrose (50)	YE (1)	NaCl (0.1), K_2_HPO_4_ (0.1), MgSO_4_ (0.1)	Shaking (–)	28	5.0	7	42.24	Yadav et al. (2014)
*Botryosphaeria rhodina* MMLR	Sucrose (50)	NH_4_NO_3_ (2)	VMSM*	Shaking (180)	28	–	3	1.80	Barbosa et al. (2003) and Vasconcelos et al. (2008)
*Botryosphaeria rhodina* MMGR	Sucrose (50)	NH_4_NO_3_ (2)	VMSM*	Shaking (180)	28	–	3	1.50	
*Botryosphaeria rhodina* MMMFR	Sucrose (50)	NH_4_NO_3_ (2)	VMSM*	Shaking (180)	28	–	3	0.40	
*Botryosphaeria rhodina* MMPI	Sucrose (50)	NH_4_NO_3_ (2)	VMSM*	Shaking (180)	28	–	3	1.30	
*Botryosphaeria rhodina* DABAC-P82	Glucose (30)	NaNO_3_ (2), YE (1)	KH_2_PO_4_ (1), MgSO_4_·7H_2_O (0.5), KCl (0.5)	Shaking (150)	28	3.7	1	17.70	Selbmann et al. (2003)
*Cordyceps sinensis* Cs-HK1	Sucrose (40)	YE (10), peptone (5)	KH_2_PO_4_ (1), MgSO_4_·7H_2_O (0.5)	Shaking (150)	20	6.8	7	1.02	Leung et al. (2009) and Huang et al. (2013)
*Fusarium coccophilum* BCC2415	Glucose (–)	PE in PDB	–	Shaking (150)	25	–	21	2.83	Madla et al. (2005)
*Fusarium oxysporum* JN604549	Glucose (20)	YE (3), peptone (5), ME (3)	KH_2_PO_4_ (0.5), NH_4_Cl (0.5), sea salt (24.4)	Fermentation	25	6.0–6.5	40	0.59	Chen et al. (2015)
*Fusarium oxysporum* Dzf17	Glucose (50)	Peptone (13)	KH_2_PO_4_ (0.6), MgSO_4_·7H_2_O (0.2), NaCl (0.6)	Shaking (150)	25	–	14	0.21	Li et al. (2014)
*Fusarium oxysporum* Y24-2	Glucose (20)	YE (3), peptone (5), NH_4_Cl (0.5)	KH_2_PO_4_ (0.5), NH_4_Cl (0.5)	Shaking (–)	25	6.0–6.5	7	0.12	Guo et al. (2010, 2013)
*Fusarium solani* SD5	Glucose (9.8), PDB	YE (0.69), PE in PDB	KH_2_PO_4_ (0.5), KCl (0.5)	Shaking (120)	28	6.46	13.7	2.28	Mahapatra and Banerjee (2012)
*Hypocreales* sp. NCHU01	Sucrose (10.6)	YE (10.92)	KH_2_PO_4_ (1), MgSO_4_ (1)	Shaking (100)	25	6.5	5	1.33	Yeh et al. (2014)
*Morchella crassipes*	Maltose (44.8)	Tryptone (4.21)	–	Shaking (150)	28	6.0	7	9.67	He et al. (2012)
*Penicillium commune*	Mannitol (20), Maltose (20), Glucose (10)	MG (10), corn syrup (1), YE (3)	KH_2_PO_4_ (0.5), MgSO_4_·7H_2_O (0.3), CaCO_3_ (20), sea salt (33)	Fermentation	28	6.5	10	0.43	Chen et al. (2013c)
*Penicillium griseofulvum*	Maltose (20), Glucose (10), Mannitol (20)	MG (10), YE (3), maize paste (1)	KH_2_PO_4_ (0.5), MgSO_4_ (0.3)	Static	20	6.5	30	0.25	Chen et al. (2013b)
*Penicillium vermiculatum* CCM F-276	Glucose (90)	NaNO_3_ (2)	KH_2_PO_4_ (1), KCl (0.5), MgSO_4_·7H_2_O (0.5), FeSO_4_·7H_2_O (0.01), CuSO_4_·5 H_2_O (0.025)	Shaking (–)	28	6.3	7	4.8	Kogan et al. (2002)
*Phoma herbarum* CCFEE 5080	Sorbitol (60)	NaNO_3_ (3)	KCl (0.5), KH_2_PO_4_ (1), MgSO_4_·7H_2_O (0.5)	Shaking (150)	28	4.7	4	13.6	Selbmann et al. (2002)
*Trichoderma pseudokoningii*	Glucose (–)	PE (–)	–	Shaking (180)	28	–	10	–	Huanga et al. (2012)
Basidiomycota									
*Agaricus nevoi* HAI 610	Mannitol (20)	Corn steep liquor (20 mM), YE (3)	KH_2_PO_4_ (0.8), Na_2_HPO_4_ (0.4), MgSO_4_·7 H_2_O (0.5)	Shaking (150)	25	6.0	5	3.00	Elisashvili et al. (2009)
*Antrodia camphorate*	Glucose (25)	Peptone (5), ME (3), YE (3)	KH_2_PO_4_·1 H_2_O (1), MgSO_4_·7 H_2_O (1)	Batch fermentation (300)	28	5.0	14	0.12	Shu and Lung (2004)
*Antrodia cinnamomea* BCRC 35396	Glucose (50)	ME (3), YE (3), calcium nitrate (5)	KH_2_PO_4_·1 H_2_O (1), MgSO_4_·7 H_2_O (1)	Shaking (150)	28	5.5	14	0.58	Lin and Sung (2006)
*Auricularia auricula* AA-2	Glucose (40)	Soybean powder (7)	KH_2_PO_4_ (4), MgSO_4_·7 H_2_O (2)	Batch fermentation (200)	28	5.4	4	7.50	Wu et al. (2006)
*Clavariadelphus truncates*	PDB (24)	ME (10), peptone (1)	–	Shaking (150)	25	–	7	–	Demir and Yamaç (2008)
*Cerrena unicolor*	PDB (24)	ME (10), peptone (1)	–	Shaking (150)	25	–	7	–	Demir and Yamaç (2008)
*Cerrena maxima* IBB 681	Maltose (20)	YE (3), (NH_4_)_2_SO_4_ (2)	KH_2_PO_4_ (0.8), Na_2_HPO_4_ (0.4), MgSO_4_·7 H_2_O (0.5)	Shaking (150)	25	6.0	5	1.20	Elisashvili et al. (2009)
*Coprinus comatus*	PDB (24)	ME (10), peptone (1)	–	Shaking (150)	25	–	7	–	Demir and Yamaç (2008)
*Cryptococcus laurentii* DSMZ 70766	Sucrose (35)	YE (3)	Na_2_HPO_4_·12 H_2_O (2.5), MgSO_4_·7 H_2_O (0.5)	Fermentation (150)	25	6.0	6	4.3	Smirnou et al. (2014)
*Cryptococcus neoformans* ATCC 24067	Glucose (2.7)	Glycine (1), thiamine (0.001)	MgSO_4_ (1.2), KH_2_PO_4_ (4)	–	30	5.5	–	–	Frases et al. (2008)
*Flammulina velutipes* SF-06	Glucose (10)	Potato (cut into pieces) (200)	KH_2_PO_4_ (1.5), MgSO_4_·7 H_2_O (1)	Batch fermentation	25	–	7	–	Ma et al. (2015)
*Fomes fomentarius*	Glucose (60)	Silkworm chrysalis (15), YE (3)	KH_2_PO_4_ (1), MgSO_4_·7 H_2_O (0.8), CaCl_2_ (0.5)	Shaking (150)	25	6.0	8	3.64	Chen et al. (2008a, b)
*Ganoderma applanatum* KFRI 646	Glucose or maltose (60)	YE (2), glutamic acid (1)	KH_2_PO_4_ (2), MgSO_4_ (0.5)	Shaking (100)	25	4.5	12	1.35	Lee et al. (2007)
*Ganoderma carnosum*	PDB (24)	ME (10), peptone (1)	–	Shaking (150)	25	–	7	–	Demir and Yamaç (2008)
*Ganoderma lucidium* UF20706	Glucose (17.5)	Peptone (2.5), wheat grains, extract (–)	KH_2_PO_4_ (1.5), MgSO_4_·7 H_2_O (1)	Shaking (150)	25	4.0	14	0.53	Fraga et al. (2014)
*Ganoderma lucidum* CCGMC 5.616	Lactose (35)	YE (5), Peptone (5)	KH_2_PO_4_ (1), MgSO_4_·7 H_2_O (0.5)	Fed-batch fermentation (–)	30	–	14	1.25	Tang and Zhang (2002)
*Ganoderma lucidium* HAI 447	Glucose (20)	YE (3), (NH_4_)_2_SO_4_ (2)	KH_2_PO_4_ (0.8), Na_2_HPO_4_ (0.4), MgSO_4_·7 H_2_O (0.5)	Shaking culture (150)	25	6.0	5	1.6	Elisashvili et al. (2009)
*Grifola frondosa*	Glucose (40)	YE (8) Corn steep powder (12) (NH_4_)_2_SO_4_ (1)	MSS**	Fed-batch fermentation (250)	25	5.0	13	3.88	Shih et al. (2008)
*Inonotus levis* HAI 796	Glucose (20)	Corn steep liquor (20 mM) YE (3)	KH_2_PO_4_ (0.8), Na_2_HPO_4_ (0.4), MgSO_4_·7 H_2_O (0.5)	Shaking (150)	25	6.0	5	3.00	Elisashvili et al. (2009)
*Laetiporus sulphureus*	PDB (24)	ME (10), peptone (1)	–	Shaking (150)	25	–	7	–	Demir and Yamaç (2008)
*Lentinus edodes*	Beer wort (7° of Balling’s scale)	–	–	Batch fermentation (150)	25	–	4	4.00	Lobanok et al. (2003)
*Lentinus strigosus*	PDB (24)	ME (10), peptone (1)	–	Shaking (150)	25	–	7	–	Demir and Yamaç (2008)
*Lenzites betulina*	PDB (24)	ME (10), peptone (1)	–	Shaking (150)	25	–	7	–	
*Phellinus gilvus*	Glucose (30)	Corn steep powder (5)	KH_2_PO_4_ (0.68), K_2_HPO_4_ (0.87), MgSO_4_·7 H_2_O (1.23)	Batch fermentation	30	4.0	11	5.30	Hwang et al. (2003)
*Phellinus igniarius* HAI 795	Maltose (20)	YE (3), (NH_4_)_2_SO_4_ (2)	KH_2_PO_4_ (0.8), Na_2_HPO_4_ (0.4), MgSO_4_·7 H_2_O (0.5)	Shaking (150)	25	6.0	5	1.80	Elisashvili et al. (2009)
*Phellinus* sp. P0988	Glucose (50)	Glutamic acid (4), (NH_4_)_2_SO_4_ (4)	KH_2_PO_4_ (1), MgSO_4_ (1)	Batch fermentation (–)	28	6.5	5	10.90	Ma et al. (2014)
*Pleurotus dryinus* IBB 903	Glucose (20)	YE (3), (NH_4_)_2_SO_4_ (2)	KH_2_PO_4_ (0.8), Na_2_HPO_4_ (0.4), MgSO_4_·7 H_2_O (0.5)	Shaking (150)	25	6.0	5	1.10	Elisashvili et al. (2009)
*Pleurotus pulmonarius*	Galactose (30)	YE (4)	KH_2_PO_4_ (1), MgSO_4_·7 H_2_O (0.2)	Shaking (120)	22	–	18	0.42	Smiderle et al. (2012)
*Pleurotus sajor*-*caju* CCB 019	Glucose (20)	(NH_4_)_2_SO_4_ (5), YE (2), Peptone (1)	KH_2_PO_4_ (1), MgSO_4_·7 H_2_O (0.2)	Shaking (120)	30	6.5-7.0	–	–	Telles et al. (2011)
*Pleurotus sajor*-*caju*	Glucose (20)	(NH_4_)_2_SO_4_ (2.5), peptone (1)	KH_2_PO_4_ (1), MgSO_4_·7 H_2_O (0.2), CaCO_3_ (1)	Batch fermentation (300)	30	4.0	20	0.94	Silveira et al. (2015)
*Polyporus arcularius*	PDB (24)	ME (10), peptone (1)	–	Shaking (150)	25	–	7	–	Demir and Yamaç (2008)
*Trametes versicolor*	Fructose (20)	YE (2), peptone (1), (NH_4_)_2_SO_4_ (5)	KH_2_PO_4_ (1), MgSO_4_·7 H_2_O (0.2)	Shaking (–)	27	6.0	7	8.00	Bolla et al. (2010)
*Trametes versicolor* IBB 897	Maltose (20)	YE (3), (NH_4_)_2_SO_4_ (2)	KH_2_PO_4_ (0.8), Na_2_HPO_4_ (0.4), MgSO_4_·7 H_2_O (0.5)	Shaking (150)	25	6.0	5	1.40	Elisashvili et al. (2009)

*ME* malt extract, *MG* monosodium glutamate, *YE* yeast extract, *PDB* potato dextrose broth, *PE* potato extract, *VMSM* vogel minimum salt medium, *MSS* mineral salt solution

VMSM* composition: Na_3_ citrate—2.5 g l^−1^, KH_2_PO_4_—5.0 g l^−1^, NH_4_NO_3_—2.0 g l^−1^, MgSO_4_·7H_2_O—0.2 g l^-1^, CaCl_2_·2H_2_O—0.1 g l^−1^, biotin—25 mg l^−1^, citric acid—5.0 mg l^−1^, ZnSO_4_·7H_2_O—5.0 mg l^−1^, Fe(NH_4_)_2_(SO_4_)_2_·6H_2_O—1.0 mg l^−1^, CuSO_4_·5H_2_O—0.25 mg l^−1^, MnSO_4_·H_2_O—0.05 mg l^−1^, H_3_BO_3_—0.05 mg l^−1^, NaMoO_4_·2H_2_O—0.05 mg l^−1^; MSS* composition: MgSO_4_·7H_2_O—1.20 g l^−1^, NaCl—0.06 g l^−1^, KH_2_PO_4_—0.20 g l^−1^, CaCl_2_—0.20 g l^−1^, FeSO_4_·7H_2_O—0.10 g l^−1^, ZnCl_2_—0.018 g l^−1^

The growth conditions of the fungus e.g. the type of culture, temperature, pH value, C and N source, appropriate salt content, and time of culture are essential for the type and amount of EPS obtained. The maximal yield of EPS produced by Ascomycota and Basidiomycota fungi was in the range from 0.12 to 42.24 g l^−1^ and depended mainly on the tested strain and culture conditions used (Table 1). The production of fungal EPS was reached in shaking cultures with a constant supply of oxygen. Bolla et al. (2010) examined production of EPS by *Trametes versicolor* and reported that the yield of EPS in shaking cultures was 8.0 g l^−1^. In turn, also the batch fermentation technique for EPS production (10.92 g l^−1^) by *Phellinus* sp. PO988 was very effective among Basidiomycota strains (Ma et al. 2014). The greatest efficiency in EPS production, i.e. up 42.24 g l^−1^, was reported by Yadav et al. (2014) in cultures of the Ascomycota fungus *Aureobasidium pullulans* RYLF-10. The cultivation time is also an important factor affecting EPS production and depends mainly on the type of culture and the type of the fungal strain. The optimal time for EPS synthesis in cultures of Ascomycota and Basidiomycota strains usually ranges from 3 to 40 days (Table 1). It is known that the most effective EPS synthesis occurs at the end of the logarithmic growth phase or early stationary phase. In contrast to bacteria, Ascomycota and Basidiomycota fungi usually require a long incubation time to produce more EPS although Wu et al. (2006) showed that *Auricularia auricula* produced relatively large amounts of EPS (7.5 g l^−1^) after 4 days of growth and Ascomycota *Botryosphaeria rhodina* strain DABAC-P82 synthesized up to 17.7 g l^−1^ of EPS after only 1 day of culture (Selbmann et al. 2003). An excessively long cultivation time contributed to reduction of EPS amounts in the liquid medium and was related to formation of low molecular EPS (Shu and Lung 2004). For example, Chen et al. (2015) obtained only 0.59 g l^−1^ of EPS after 40 days of *Fusarium oxysporum* JN604549 cultivation. Ascomycota and Basidiomycota strains produced amounts of maximum EPS at a temperature range between 20 and 30 °C. In turn, the pH values of the culture medium are usually acidic or neutral and oscillate around a range of 4.0–7.0 for maximum EPS production. It was observed that the pH values varied during the cultivation days. For example, *B. rhodina* DABAC-P82 strains changed the initially acidic pH value from 3.7 to 5.9–6.5 after 1 day of cultivation (Selbmann et al. 2003). The EPS production efficiency depends on the type of the C source. The main source of C in most Ascomycota and Basidiomycota cultures was glucose, but potato dextrose broth (PDB) and sugars such as sucrose, maltose, mannitol, lactose, and fructose were used as well. The concentration of the compound serving as a C source is an additional important factor in the synthesis of EPSs. It has been shown that the most optimal concentration of the C source usually varies between 30 and 50 g l^−1^ (Table 1). Another important factor in the production of EPS in fungi is the presence of organic N sources (e.g. corn steep liquor, yeast extract, peptone, malt extract, soybean powder) and inorganic N sources comprising ammonium sulfate, sodium nitrate, and ammonium chloride, as well as mineral salts in the growth medium (Table 1). The highest yield (10.9 g l^−1^) of Basidiomycota EPS was obtained in the *Phellinus* sp. P0988 culture with glutamic acid and ammonium sulfate (4 g l^−1^) used as the main N sources and glucose (50 g l^−1^) as the main C source (Ma et al. 2014). The highest yield (42.2 g l^−1^) of Ascomycota EPS was described for *Aureobasidium pullulans* culture with yeast extract (1 g l^−1^) and sucrose (50 g l^−1^) as the main N and C sources, respectively (Yadav et al. 2014).

In cultures of Ascomycota *B.rhodina* strain, a Vogel Minimum Salt Medium (VMSM) with ammonium nitrate as the main N source but also with biotin and traces (1.0 mg l^−1^) of ammonium ferrous sulfate was used (Barbosa et al. 2003; Vasconcelos et al. 2008). This VMSM and mineral salt solution (MSS) used in Basidiomycota *Grifola frondosa* strain culture was a very rich source of different salts (Table 1). Phosphorous and magnesium are components of almost all cultures used for derivation of fungal EPSs and seem a most important mineral additives stimulating EPS production. Such compounds as potassium dihydrogen phosphate (KH_2_PO_4_) and dipotassium hydrogen phosphate (K_2_HPO_4_) are most effectively used by fungi as a phosphorus source. In addition, magnesium sulfate (MgSO_4_·7H_2_O), sodium phosphate (Na_2_HPO_4_), sodium chloride, and potassium chloride are usually used for supplementation of media for cultivation of EPS producers.

### EPSs isolation, extraction, and purification methods

In contrast to cell-wall or cytosolic PSs, EPSs do not require drastic methods of extraction with hot water or organic solvents (Madla et al. 2005; Chen et al. 2008a, b; Mahapatra and Banerjee 2013a). Usually, to obtain crude EPS, the culture fluid is precipitated by alcohols such as ethanol (absolute or 95 %) or methanol and only in some cases by acetone or isopropanol at a temperature of 4 °C for a period of 12–24 h (Table 2). The main extraction method of EPS obtaining from Ascomycota and Basidiomycota strains cultures is the precipitation with 95 % ethanol used in a proportion 1/4 (v/v) of supernatant and ethanol. Particularly in Ascomycota strains, primary purification of supernatant with 5 % trichloroacetic acid (TCA) is sometimes applied (Yadava et al. 2012). After dialysis against water, crude EPSs are generally stored as vacuum-dried or lyophilized powder. The next step of EPS purification is deproteinization thereof using Sevage reagent (Chen et al. 2011, 2013a, b, c). The main methods used for purification of Ascomycota and Basidiomycota EPSs are Ion Exchange Chromatography (IEC) and a type of Size Exclusion Chromatography (SEC)–Gel Permeation Chromatography (GPC) (Table 2) (Shu and Lung 2004; Chen et al. 2011; Ma et al. 2015).

**Table 2:** EPS of Ascomycota and Basidiomycota strains—isolation, extraction, and purification methods

EPS producer	The main steps during the procedure of				EPS characteristics				References
	Isolation		Purification		Solubility	Compositions	Linkage type	M_w_ (kDa)	
	Precipitation method (supernatants/alcohol proportion v/v)	Period of dialysis against water (h)	EPS solution	Chromatographic methods					
Ascomycota									
*Alternaria alternate*	95 % ethanol (1/5), 16 h, 4 °C	–	–	–	–	–	–	–	Nehad and El-Shamy (2010)
*Aspergillus* sp. Y16	95 % ethanol (1/3)	48	Water	IEC GPC	Water	EPS1-Man, Gal EPS2-Man, Glc	EPS1—1 → 2;1 → 6, EPS2—1 → 3; 1 → 6	EPS1-15.0 EPS2-6.0	Chen et al. (2011)
*Aspergillus* sp. RYLF17	95 % ethanol (1/4), 24 h, 4 °C	–	–	–	–	–	–	–	Yadava et al. (2012)
*Aspergillus versicolor*	95 % ethanol (1/3)	48	Water	IEC GPC	Water	Glc, Man	α-1 → 6	500.0	Chen et al. (2013a)
*Aureobasidium pullulans* RYLF-10	95 % ethanol (1/4), 24 h, 4 °C	–	Water	–	–	–	–	–	Yadav et al. (2014)
*Botryosphaeria rhodina* MMLR	Absolute ethanol (1/3)	48		GPC	Water, 1 M NaOH	Glc	β-1 → 6	–	Barbosa et al. (2003) and Vasconcelos et al. (2008)
*Botryosphaeria rhodina* MMGR	Absolute ethanol (1/3)	48		GPC	Water, 1 M NaOH	Glc	β-1 → 6	–	
*Botryosphaeria rhodina* MMPI	Absolute ethanol (1/3)	48		GPC	Water, 1 M NaOH	Glc	β-1 → 6	–	
*Botryosphaeria rhodina* MMMFR	Absolute ethanol (1/3)	48	Water	GPC	Water, 1 M NaOH	Glc	β-1 → 3 β-1 → 6	–	
*Botryosphaeria rhodina* DABAC-P82	Ethanol (1/2), 16 h, 4 °C, repeated twice	24	Water	GPC	Water	Glc	β-1 → 6 β-1 → 3	4.9	Selbmann et al. (2003)
*Cordyceps sinensis* Cs-HK1	95 % ethanol (5 ratios: 1/0.2, 1/0.4, 1/1, 1/2, 1/5)	–	–	–	–	Glc	–	EPS_0.2_-47,400.0 EPS_0.4_-47,400.0 EPS_1_-253,000.0 EPS_2_-630.0 EPS_5_-16.0	Leung et al. (2009) and Huang et al. (2013)
*Fusarium coccophilum* BCC2415	95 % ethanol (1/4), 12 h, −20 °C	–	Water	GPC	Water	–	–	2.8	Madla et al. (2005)
*Fusarium oxysporum* JN604549	95 % ethanol (1/3)	72	Water	IEC GPC	Water	Gal, Glc, Man	β-1 → 6 β-1 → 2	61.2	Chen et al. (2015)
*Fusarium oxysporum* Dzf17	95 % ethanol (1/3), 48 h, 4 °C	–	Water	–	Water	–	–	–	Li et al. (2014)
*Fusarium oxysporum* Y24-2	95 % ethanol (1/4)	72	Water	IEC GPC	Water	Gal, Glc	α-1 → 2 β-1 → 6	36.0	Guo et al. (2010, 2013)
*Fusarium solani* SD5	Absolute ethanol (1/5), 24 h, 4 °C	24	Water	GPC	Water	Gal, Rha	α-1 → 2 β-1 → 4 α-1 → 6	187.0	Mahapatra and Banerjee (2012)
*Hypocreales* sp. NCHU01	95 % ethanol (1/4)	–	–	–	1 M NaOH 60 °C, 1 h	–	–	–	Yeh et al. (2014)
*Morchella crassipes*	95 % ethanol (1/4), 16 h, 4 °C	–	0.2 M NaCl	GPC	0.2 M NaCl	–	–	19.6	He et al. (2012)
*Penicillium commune*	95 % ethanol (1/3)	48	Water	IEC GPC	Water	Glc, Man, Gal	α-1 → 2 β-1 → 6	18.3	Chen et al. (2013c)
*Penicillium griseofulvum*	95 % ethanol (1/3)	48	Water	IEC GPC	Water	Man, Gal	α-1 → 2 β-1 → 5 α-1 → 6	20.0	Chen et al. (2013b)
*Penicillium vermiculatum* CCM F-276	Ethanol (1/4) repeated three times	–	Water	GPC	Water	Glc, Gal, Man	β-1 → 6 β-1 → 3	77.0	Kogan et al. (2002)
*Phoma herbarum* CCFEE5080	Absolute ethanol (1/2), 16 h, 4 °C, repeated twice	24	Water	GPC	Water	Glc	β-1 → 3 α-1 → 6	7.4	Selbmann et al. (2002)
*Trichoderma pseudokoningii*	95 % ethanol (1/3), 16 h, 4 °C	–	Water	IEC GPC	Water	Rha, Xyl, Fuc, Man, Glc, Gal	–	31.9	Huanga et al. (2012)
Basidiomycota									
*Agaricus nevoi* HAI 610	95 % ethanol (1/3), 12 h, 20 °C	–	–	–	–	–	–	–	Elisashvili et al. (2009)
*Antrodia camphorata*	–	48	Water	GPC	Water	–	–	EPS1 < 50 EPS2 > 50 < 400 EPS3 > 400	Shu and Lung (2004)
*Antrodia cinnamomea* BCRC 35396	95 % ethanol (1/4), 12 h, 4 °C, repeated twice	–	–	–	–	–	–	–	Lin and Sung (2006)
*Auricularia auricula* AA-2	95 % ethanol, 12 h, 4 °C, repeated twice	–	–	–	–	–	–	–	Wu et al. (2006)
*Clavariadelphus truncates*	95 % ethanol (1/4), 12 h, 4 °C	–	–	–	–	–	–	–	Demir and Yamaç (2008)
*Cerrena unicolor*	95 % ethanol (1/4), 12 h, 4 °C	–	–	–	–	–	–	–	Demir and Yamaç (2008)
*Cerrena maxima* IBB 681	95 % ethanol (1/3), 12 h, 20 °C	–	–	–	–	–	–	–	Elisashvili et al. (2009)
*Corpinus comatus*	95 % ethanol (1/4), 12 h, 4 °C	–	–	–	–	–	–	–	Demir and Yamaç (2008)
*Cryptococcus laurentii* DSMZ 70766	Absolute isopropanol (1/3)	–	–	–	–	Man, GlcA, Glc, Gal, Xyl	–	2000.0	Smirnou et al. (2014)
*Cryptococcus neoformans* ATCC 24067	95 % ethanol (1/3), CTAB-PS	24	Water	–	Water	Xyl, GlcA, Man, Gal, Glc	–	1210.0	Frases et al. (2008)
*Flammulina velutipes* SF-06	95 % ethanol (1/3), 24 h, 4 °C	10	Water	ICE GPC	–	EPS1-Rha, Glc EPS2-Rha, Gal	–	–	Ma et al. (2015)
*Fomes fomentarius*	95 % ethanol (1/4), 12 h, 4 °C	–	–	–	–	–	–	–	Chen et al. (2008a, b)
*Ganderma applanatum* KFRI 646	95 % ethanol (1/4), 12 h, 4 °C	72	–	–	Water	Glc, Gal, Man, Xyl	–	EPS1-2300.0 EPS2-1038.0	Lee et al. (2007)
*Ganoderma carnosum*	95 % ethanol (1/4), 12 h, 4 °C	–	–	–	–	–	–	–	Demir and Yamaç (2008)
*Ganoderma lucidium* UF20706	95 % ethanol (1/4), 12 h, 4 °C	–	–	HPAEC	–	Fuc, Gal, Glc, Man	1 → 3; 1 → 4	–	Fraga et al. (2014)
*Ganoderma lucidum* CCGMC 5.616	95 % ethanol (–), 12 h, 4 °C	–	–	–	Water, NaOH (60 °C)	–	–	–	Tang and Zhang (2002)
*Ganoderma lucidium* HAI 447	95 % ethanol (1/3), 12 h, 20 °C	–	–	–	–	–	–	–	Elisashvili et al. (2009)
*Grifola frondosa*	95 % ethanol (1/4), 12 h, 4 °C	–	–	GPC	1 M NaOH (60 °C)	–	–	EPS1-390,840.0 EPS2-3097.0	Shih et al. (2008)
*Inonotus levis* HAI 796	95 % ethanol (1/3), 12 h, 20 °C	–	–	–	–	–	–	–	Elisashvili et al. (2009)
*Laetiporus sulphurous*	95 % ethanol (1/4), 12 h, 4 °C	–	–	–	–	–	–	–	Demir and Yamaç (2008)
*Lentinus edodes*	95 % ethanol (1/1), 12 h, 4 °C	–	–	–	Water (poor soluble) 0.5 % NaOH	–	–	–	Lobanok et al. (2003)
*Lentinus strigosus*	95 % ethanol (1/4), 12 h, 4 °C	–	–	–	–	–	–	–	Demir and Yamaç (2008)
*Lenzites betulina*	95 % ethanol (1/4), 12 h, 4 °C	–	–	–	–	–	–	–	
*Phellinus gilvus*	95 % ethanol (1/4), 12 h, 4 °C	–	Buffer, pH 6.8	SEC/MALS	Water	Mal, Arab, Xyl, Man, Gal, Glc	–	EPS1–8 628.0 EPS2–1 045.0 EPS3–610.9 EPS4–335.5	Hwang et al. (2003)
*Phellinus igniarius* HAI 795	95 % ethanol (1/3), 12 h, 20 °C	–	–	–	–	–	–	–	Elisashvili et al. (2009)
*Phellinus* sp. P0988	95 % ethanol (1/3), 12 h, 4 °C	–	2 % tannic acid	–	Water	–	–	–	Ma et al. (2014)
*Pleurotus dryinus* IBB 903	95 % ethanol (1/3), 12 h, 20 °C	–	–	–	–	–	–	–	Elisashvili et al. (2009)
*Pleurotus pulmonarius*	95 % ethanol (1/3)	+	–	IEC	–	Man, Gal, Glc 3-*O*-methyl-Gal	1 → 3; 1 → 6	–	Smiderle et al. (2012)
*Pleurotus sajor*-*caju* CCB 019	Acetone (1/3), 24 h, 4 °C	–	–	–	–	Man, Gal, Glc 3-*O*-methyl-Gal	–	–	Telles et al. (2011)
*Pleurotus sajor*-*caju*	95 % ethanol		–	HPAEC	–	Man, Gal 3-*O*-methyl-Gal	1 → 6	64.0	Silveira et al. (2015)
*Polyporus arcularius*	95 % ethanol (1/4), 12 h, 4 °C	–	–	–	–	–	–	–	Demir and Yamaç (2008)
*Trametes versicolor*	Isopropanol (1/1), 24 h, 4 °C	–	–	–	–	–	–	–	Bolla et al. (2010)
*Trametes versicolor* IBB 897	Absolute ethanol (1/3), 12 h	–	–	–	–	–	–	–	Elisashvili et al. (2009)

Arab, arabinose; Fuc, fucose; Gal, galactose; Glc, glucose; GlcA, glucuronic acid; Mal, maltose; Man, mannose; Rha, rhamnose; Xyl, xylose; GPC, Gel Permeation Chromatography type of Size Exclusion Chromatography (SEC); HPAEC, High Performance Anion Exchange Chromatography; IEC, Ion Exchange Chromatography; SEC/MALS, the combination of SEC with Multi-Angle Light Scattering Analysis (MALS); CTAB-PS, isolation of EPS by cetyltrimethylammonium bromide precipitation

### EPS characteristics

Ascomycota and Basidiomycota EPSs are usually soluble in water or in a NaCl solution but data concerning the degree of EPS solubility in water are limited. In some cases, solubility of EPSs in alkali solutions (mainly 1 M NaOH) was determined (Table 2). The chemical structures and properties of EPS such as the monosaccharide composition, the linkage type, the molecular weight, or the conformation chain were usually evaluated by different experimental analyses including chromatography technology such as TLC, HPLC, and GLC as well as spectrum analysis such as FTIR and 1D and 2D NMR spectroscopy or GLC-MS (Kozarski et al. 2012; Osińska-Jaroszuk et al. 2014; Silveira et al. 2015). Ascomycota and Basidiomycota EPSs are mainly heteropolysaccharides but in the case of homopolysaccharides glucose is their only monomer (Table 2). The sugar content of various EPSs is varied but glucose, mannose, galactose, xylose, fucose, and rhamnose monomers have frequently been found in fungal EPSs. Moreover, individual monosaccharides synthesized by different groups of Ascomycota and Basidiomycota fungi had different molecular weight (Table 2). EPSs isolated from the Ascomycota and Basidiomycota strains have a different linkage pattern but a 1 → 6 linkage type is dominant, and presence of different types of linkage has been described for Ascomycota EPSs. Guo et al. (2013) reported that EPS from *Fusarium oxysporum* Y24-2 consists of a disaccharide repeating units with the following structure (n ≈ 111): [→2)-β-d-Galf(1 → 6)-α-d-Glcp(1→](n). A homogenous EPS from *Aspergillus versicolor* is mainly composed of (1 → 6)-linked α-d-glucopyranose residues, slightly branched by single α-d-mannopyranose units attached to the main chain at C-3 positions of the glucan backbone (Chen et al. 2013a). *B. rhodina* MMGR produces EPS containing β-1 → 6 branched glucose residues (Barbosa et al. 2003; Vasconcelos et al. 2008). *Penicillium griseofulvum* produces an EPS composed of a long chain of galactofuranan and a mannose core. The galactofuranan chain consists of (1 → 5)-linked β-galactofuranose, with additional branches at C-6 consisting of (1→)-linked β-galactofuranose residues and phosphate esters. The mannan core is composed of (1 → 6)-linked α-mannopyranose substituted at C-2 by (1→)-linked α-mannopyranose residues, disaccharide, and trisaccharide units of (1 → 2)-linked α-mannopyranose (Chen et al. 2013b). There are only few studies on the linkage type of EPS isolated from Basidiomycota. Silveira et al. (2015) reported that the EPS produced by edible mushrooms *Pleurotus sajor*-*caju* was a mannogalactan composed of mannose (37.0 %), galactose (39.7 %), and 3-*O*-methyl-galactose (23.3 %) and comprised a main chain of (1 → 6)-linked α-d-Galp and 3-*O*-methyl-α-d-Galp units. *Ganoderma lucidium* produces EPSs that are mainly β-(1 → 4)-linked branched glucans containing (1 → 3) linkages as well (Fraga et al. 2014).

### Bioactive properties and potential biotechnological (medical, environmental, and agricultural) applications of EPS

Many bioactive properties of Ascomycota and Basidiomycota EPSs such as antioxidative, antimicrobial, immunomodulatory, antitumor, hypolipidemic, hypoglycemic, and hepatoprotective activity can find medical applications. The activities of fungal EPSs in such processes as mineral solubilization, heavy metal sorption and hydrocarbon removal as well as eliciting plant resistance created a possibility of potential environmental and agricultural applications of these EPSs in biofertilization, soil/water bioremediation, and plant bioprotection (Table 3). The bioactive properties of PSs are known to depend on many factors, including the structure, monosaccharide components, molecular mass, conformation, configuration of glycosidic bonds, and extraction and isolation methods (Zhou and Chen 2011).

**Table 3:** Examples of bioactive properties and potential medical, environmental, and agricultural applications of EPSs obtained from selected Ascomycota and Basidiomycota strains

Bioactivities	Ascomycota		Basidiomycota	
	EPS producer	References	EPS producer	References
*Potential medical applications*				
Antioxidative	*Aspergillus* sp. Y16	Chen et al. (2011)	*Agaricus brasiliensis*	Kozarski et al. (2011)
	*Cordyceps sinensis*	Leung et al. (2009)	*Ganoderma applanatum*	Kozarski et al. (2012)
	*Fusarium oxysporum*	Chen et al. (2015)	*Ganoderma lucidium*	Jia et al. (2009), Liu et al. (2010) and Kozarski et al. (2012)
	*Morchella crassipes*	He et al. (2012)	*Lentinus edodes*	Kozarski et al. (2012)
			*Trametes versicolor*	Kozarski et al. (2012)
			*Phellinus* sp. P0988	Ma et al. (2014)
			*Flammulina velutipes* SF-06	Ma et al. (2015)
Antimicrobial	*Hirsutella* sp.	Li et al. (2010)	*Cerrena unicolor*	Demir and Yamaç (2008)
			*Ganoderma applanatum*	Osińska-Jaroszuk et al. (2014)
			*Ganoderma carnosum*	Demir and Yamaç (2008)
			*Lenzites betulina*	Demir and Yamaç (2008)
			*Polyporus arcularius*	Demir and Yamaç (2008)
Immunomodulatory	*Fusarium coccophilum* BCC2415	Madla et al. (2005)	*Ganoderma applanatum*	Osińska-Jaroszuk et al. (2014)
	*Cordyceps sinensis*	Zhang et al. (2010)	*Pleurotus sajor-caju*	Silveira et al. (2015)
			*Cryptococcus* *neoformans*	Frases et al. (2008)
Antitumor	*Trichoderma pseudokoningii*	Huanga et al. (2012)	*Agaricus blazei*	Yu et al. (2009)
	*Cordyceps militaris*	Kim et al. (2010)	*Fomes fomentarius*	Chen et al. (2008a, b)
	*Cordyceps sinensis*	Zhang et al. (2010)	*Ganoderma applanatum*	Osińska-Jaroszuk et al. (2014)
			*Ganoderma tsugae*	Hsu et al. (2008)
			*Pleurotus sajor-caju* CCB 019	Telles et al. (2011)
Wound management			*Cryptococcus* *laurentii*	Smirnou et al. (2014)
Hypoglycemic	*Cordyceps militaris*	Kim et al. (2001)	*Tremella fuciformis*	Cho et al. (2007)
	*Lentinus edodes*	Yang et al. (2002)	*Phellinus baumii*	Cho et al. (2007)
	*Botryosphaeria rhodina*	Miranda-Nantes et al. (2011)	*Lentinus edodes*	Kim et al. (2001)
			*Phellinus linteus*	Kim et al. (2001)
			*Cerrena unicolor*	Kim et al. (2001)
			*Coprinus comatus*	Yamac et al. (2009)
			*Lenzites betulina*	Yamac et al. (2009)
Hypolipidemic	*Cordyceps militaris*	Yang et al. (2000)	*Lentinus edodes*	Kim et al. (2001)
	*Botryosphaeria rhodina*	Miranda-Nantes et al. (2011)	*Phellinus linteus*	Kim et al. (2001)
Hepatoprotective			*Antrodia cinnamomea*	Ho et al. (2008)
*Potential environmental and agricultural application*				
Mineral solubilization (biofertilization)	*Penicillium* sp.	Wakelin et al. **(** 2004)	*Pleurotus ostreatus*	Seneviratne et al. (2008)
Heavy metal sorption (bioremediation)	*Aspergillus* *fumigatus*	Lian et al. (2008)		
	*Hansenula anomala* CCY 38-1-22	Breierová et al. (2002)		
	*Colletotrichum* sp.	Da Silva et al. (2014)		
	*Pestalotiopsis* sp. KCTC 8637	Moon et al. (2006)		
	*Aureobasidium pullulans* CH1	Radulović et al. (2008)		
Hydrocarbon removal (bioremediation)			*Flavodon flavus* NIOCC#312	Raghukumar et al. (2006)
Eliciting plant resistance (bioprotection)	*Fusarium oxysporum* Dzf17	Li et al. (2011)		

### Potential medical applications

Given their diversity, fungal EPSs could be used in medicine, for example as antioxidant and antimicrobial agents applied for acceleration of wound healing and fighting bacterial and viral infections as well as antitumor agents activating immune response in the host and supporting chemotherapy treatment (Zhang et al. 2002, 2007; Chen and Seviour 2007; Liu et al. 2009).

### Antioxidative and antimicrobial activities

Antioxidative properties have been described for fungal compounds, mainly represented by phenolic compounds, β-tocopherol and β-carotene, PS-protein complexes, and EPSs (Palacios et al. 2011). Until now, it has been demonstrated that ethanol extracts of nearly 150 species of fungi exhibit antioxidative activity (Chang and Miles 2004). These antioxidative properties have been recognized for several EPSs of both Ascomycota and Basidiomycota strains (Table 3). Antioxidant activity assigned to PSs consists in chelation of ferrous ions Fe^2+^ or prevention of Fenton’s reaction and inhibition of lipid peroxidation as well as enhancement of the enzymatic activity of the antioxidant system such as superoxide dismutase, catalase, or glutathione peroxidase (Kozarski et al. 2014). Antioxidants may react directly with reactive oxygen species or indirectly by reduced oxidation reaction metabolites, not allowing formation of oxygen free radicals. Therefore, PSs show greater antioxidant properties than monosaccharides because the most important factor that has an influence on the ability of EPSs to scavenge free radicals is the size of the carbohydrate molecule. The choice of a proper method of extraction of PS and PS–protein complexes, especially the type of applied solvents, also affects the antioxidant activity of purified preparations. For example, water extracts of fungal PSs contain proteins and phenol compounds with antioxidant activity (Zhou and Chen 2011; Kozarski et al. 2014).

There are many reports available describing the antibacterial properties of fungal EPSs, especially those from Basidiomycota strains from the genus *Cerrena*, *Ganoderma*, *Lenzites* and *Polyporus*, in relation to both gram-positive and gram-negative bacteria (Demir and Yamaç 2008). EPS from *Ganoderma applanatum* showed antibacterial properties against *Staphyloccoccus aureus* and a toxic effect against *Vibrio fischeri* cells (Osińska-Jaroszuk et al. 2014). For Ascomycota (*Hirsutella* strain) EPS, also antimicrobial activity against *Bacillus subtilis* and *Micrococcus tetragenus* was studied (Li et al. 2010).

### Immunomodulatory and antitumor activities and wound management

Antitumor activity of PSs is closely related to their immunomodulatory and immunostimulant activity, which is affected by many physical and chemical properties. Particles of β-(1 → 3) glucans with the molecular weight less than 20 kDa have either no or low immunomodulatory activity (Bohn and BeMiller 1995). There is a correlation between the conformation and weight of the molecule because only a molecule with molecular weight of at least 90 kDa may take a triple helix structure. There are a number of papers on the immunomodulatory and antitumor action of EPSs from both Ascomycota and Basidiomycota strains (Table 3). Lee et al. (2007) has demonstrated that the effect of EPS from *G. applanatum* on TNF-α was dependent on its molecular size. Within the range of fungal PS, the most active antitumor properties are exhibited by β-glucans binding by β-(1 → 3)-glycosidic bonds. Not without significance is also the additional share of side chains linked by β-(1 → 6) glycosidic bonds affecting the degree of branching molecules, which is reflected in increased antitumor activity of β-(1 → 3) glucan (Chen and Seviour 2007). The action of PS mainly involves stimulation of macrophages and dendritic cells (Kim et al. 2010) for production of various kinds of cytokines, including TNF-α, IFN-γ, and IL-1β, and stimulation of NK cells, T and B, which is connected with inhibition of cancer cell growth (Lindequist et al. 2005; Lee et al. 2010). After recognition and attachment of the PS molecule, immune processes such as production of free radicals, phagocytosis, or production of cytokines involved in inflammation are activated (Brown and Gordon 2005; Chan et al. 2009). Chen et al. (2008a, b) showed that EPS from *Fomes fomentarius* has a direct antiproliferative effect in vitro on SGC-7901 human gastric cancer cell. Crude EPS obtained by Osińska-Jaroszuk et al. (2014) from *G. applanatum* exhibited antitumor activity against carcinoma cells (lines SiHa) and stimulated the production of Il-6 and TNF-α by the macrophage line THP-1. EPSs produced by *Pleurotus sajor*-*caju* act as an anti-inflammatory agent reducing nociception and edema (Silveira et al. 2015). Kim et al. (2010) demonstrated that the cordlan PS isolated from *Cordyceps militaris* induced phenotypic maturation of dendritic cells demonstrated. Zhang et al. (2010) reported immunomodulatory function and antitumor activity of an EPS fraction EPSF prepared from *Cordycepssinensis*, which significantly enhanced the Neutral Red uptake capacity of peritoneal macrophages and splenic lymphocyte proliferation in B16-bearing mice and inhibited metastasis of B16 melanoma cells to lungs and livers.

EPS recovered from the culture supernatant of the human pathogenic fungus *Cryptococcusneoformans* can elicit the immunological response providing convenient source of EPS preparations suitable for immunological studies and of EPS-based vaccines for prevention of cryptococcosis (Datta and Pirofski 2006; Frases et al. 2008; Table 3).

Furthermore, EPS obtained from culture of *Cryptococcus laurentii* belonging to the same genus seems to be a very promising biotechnological product in wound management, as it significantly improved excisional wound healing in rats in in vitro experiments (Smirnou et al. 2014).

### Hypoglycemic, hypolipidemic, and hepatoprotective activities

Several PSs from edible fungi have a proven hypoglycemic effect via a decrease in the glucose level in blood or modulation of glucose/insulin metabolism. A majority of publications on this topic are related to studies of intracellular PSs (Mao et al. 2009). For example, intracellular PS obtained from *Ganoderma lucidium* decreased fasting serum glucose and insulin levels, and the epididymal fat/BW ratio in type-2 diabetic mice (Xiao et al. 2012) and delayed progression of diabetic renal complications (He et al. 2006). There are also a growing number of reports concerning the ability of some EPSs to reduce glucose levels (Table 3). Cho et al. (2007) reported that EPSs from two different Basidiomycota strains, *Tremella fuciformis* and *Phellinus baumii,* exhibited a considerable hypoglycemic effect and improved insulin sensitivity through the regulation of peroxisome proliferator-activated receptor (PPAR)-γ-mediated lipid metabolism. Oral administration of EPS produced by *Cerrena unicolor*, *Coprinus comatus*, and *Lenzites betulina* significantly decreased glucose levels in the serum in streptozotocin-induced diabetic rats (Yamac et al. 2009).

One of the pharmacological properties of fungal EPS is the ability to lower the level of cholesterol and the content of lipids in blood (Table 3). After application of glucan, increased excretion of bile acids and short chain fatty acids was observed. This process inhibits the incorporation of acetate to serum lipids, a substrate required for the synthesis of sterols and fatty acids. This is probably related to the gelling properties of glucans, which also inhibit absorption of cholesterol and triglycerols (Guillamon et al. 2010). Significant reduction of total cholesterol and triglyceride levels in plasma was observed in rats fed with *C. militaris* and *Lentinus edodes* EPSs (Yang et al. 2000, 2002). Botryosphaeran, a water-soluble EPS of the β-(1 → 3; 1 → 6)-D glucan type isolated from the culture medium of *Botryosphaeria rhodina,* administered by gavage, also reduced the plasma levels of total cholesterol and low density lipoprotein-cholesterol in hyperlipidemic rats (Miranda-Nantes et al. 2011). Hypolipidemic and hypoglycemic activities have also been described for EPSs obtained from Ascomycota strains from species *C. militaris* and *B. rhodina* cultures (Table 3).

The possible role of EPS in hepatoprotection has also been discussed. For example, the hepatoprotective activity of water extracts containing EPS from *Antrodia cinnamomea* on ethanol-induced cytotoxicity in AML12 hepatocytes was determined (Ho et al. 2008).

## Potential environmental and agricultural applications

### The EPS-as potential biofertilizers enhanced mineral solubilization

The mechanisms used by microorganisms for mineral solubilization have been attributed mainly to acidification, exchange reactions, and chelation (Yadav and Tarafdar 2003). It was found that the ability to release nutrients from minerals (weathering) by fungi resulted from the action of fungal EPS in cooperation with organic acids synthesized by fungi, causing precipitation and dissolution of soil minerals through acidification of surrounding hyphae and chelation of different ions (Welch et al. 1999; Rogers and Bennett 2004; Sheng 2005; Rosling et al. 2009; Xiao et al. 2012). Acidic metabolites adsorbed on the EPS surface bind SiO_2_ ions from silicate minerals, thereby contributing to K^+^ release in the soil solution (Liu et al. 2006). Fungal EPSs also contain their own acidic constituents, e.g. carboxylic acid, uronic acids, or functional groups enhancing mineral dissolution (Welch et al. 1999). Many saprotrophic fungi producing EPS, including strains from the genera *Penicillium* (*P. simplicissimus, P. giseofulvum, P. radicum*) and *Aspergillus* (*A. fumigatus*, *A. niger*), effectively release phosphorus and potassium through degradation of minerals e.g. Ca phosphates, Fe/Al phosphates, mica, montmorillonite, and K-feldspar and belong to a group of phosphate- or potassium-solubilizing microorganisms (PSM, KSM) (Welch et al. 1999; Wakelin et al. 2004; Barroso and Nahas 2005; Sheng 2005; Yi et al. 2008; Smits 2009; Mahapatra and Banerjee 2013b). *Penicillium* and *Aspergillus* strains are very often a fungal component of a special kind of tested (lab-stage) biofertilizers like fungal–bacterial and fungal–rhizobial biofilmed biofertilizers (FBBs and FRBs, respectively) (Wakelin et al. 2004; Lian et al. 2008). There are commercial biofertilizers with *Penicillium* strains e.g. Provide, TagTeam (produced by Philom BIOS; Canada).

EPSs play a key role in formation of biofilm facilitating colonization of soil particles, seeds, and roots systems. The degree of phosphate solubilization increased up to 230 % after the use of biofilm of *Penicillium* spp., *Pleurotus ostreatus*, and *Xanthoparmeliamexicana* in comparison to fungal monocultures (Seneviratne et al. 2008).

### EPS as a potential component of bioremediation preparations removing heavy metals and hydrocarbons

Metal biosorption by EPSs involves ionic interactions (e.g. ion exchange, electrostatic interaction) and physical entrapments (Breierová et al. 2002; Da Silva et al. 2014). EPSs possess a substantial quantity of functional groups (amine, phosphate, hydroxyl, carboxyl, and urinate), which increase the negative charge of EPSs and their ion exchange properties and flocculation activities, and can coordinate with metal ions (complexation) and form organic precipitation (Breierová et al. 2002; Moon et al. 2006; Abdel-Aziz et al. 2012). Under the presence of heavy metals, EPS is more heterogenic and contains higher amounts of different components and reactive groups than under absence of metal (Breierová et al. 2002). After purification of crude pullulan obtained from *Aureobasidium pullulans* CH1 strain, when glucose was its only component, no metal (Cu, Fe, Zn, Mn, Pb, Cd, Ni and Cr) biosorption was reported (Radulović et al. 2008). Exopolymers of *Hansenula anomala* CCY 38-1-22 bound 90 % of the total amount of Cd ions sorbed by this resistant strain, while the sensitive strain of *Saccharomyces cerevisiae* CCY 21-4-100 accumulated this metal predominantly in the cellular compartments (94 %) (Breierová et al. 2002). The biosorption of Cd and Pb ions on EPS produced by the fungus *Colletotrichum* sp. contributed to removal of 79 and 98 % of cadmium and lead, respectively, from a solution with the initial concentration of these metals of 100 mg l^−1^ (Da Silva et al. 2014). Moon et al. (2006) found that each gram of pestan (a specific EPS produced by *Pestalotiopsis* sp. KCTC 8637) absorbed 120 mg of lead or 60 mg of zinc. Fungal EPSs can be as effective in biosorption as fungal biomass (Moon et al. 2006), and can be used as potential biosorbents for removal of heavy metals from wastewaters. The interaction of a marine isolate of the white-rot fungus *Flavodon flavus* NIOCC#312 EPS with specific lignin-degrading enzymes (e.g. peroxidases and laccase) appears to help in degradation of toxic organic compounds by breaking down polycyclic aromatic hydrocarbons before their contact with mycelium (Raghukumar et al. 2006).

### EPSs as potential biocontrol preparations eliciting plant resistance

In the literature, there are many reports concerning the ability of bacterial EPSs and fungal cell wall PSs to induce plant resistance (Tamm et al. 2011) but the ability of fungal EPSs to induce resistance is still poorly studied. To our knowledge, there are only two reports about fungal EPS acting as an elicitor of systemic plant resistance (Table 3). Li et al. (2014) have shown that an oligosaccharide obtained from the EPS of the endophytic fungus *Fusarium oxysporum* Dzf17 contributed to an increase in the activity of defense-related enzymes such as phenylalanine ammonia lyase, polyphenoloxidase, and peroxidase in *Dioscorea zingiberensis* suspension cells and seedling cultures. El Oirdi et al. (2011) have shown that EPS of *Botrytis cinerea* acts as a suppressor of the jasmonic acid (JA) signaling pathway, induces accumulation of salicylic acid (SA) in tomato, and enhances resistance against the hemibiotrophic pathogen *Pseudomonas syringae*. The phenomenon of induced systemic plant resistance—systemic acquired resistance (SAR) mediated by a SA-dependent process and induced systemic resistance (ISR) mediated by JA and ethylene (ET) involves elevation of the level of phenylpropanoid pathway metabolites not only at the site of introduction of the elicitor but also in the entire plant, even in tissues distant from the infection site (Walters et al. 2013).

## Conclusion

Many research groups conduct studies on the EPSs from Ascomycota and Basidiomycota fungi, considering their potential use in various fields of science and industry. The data described above imply that the current work on fungal EPSs is the tip of the iceberg, and many exciting scientific findings are still to come. While knowledge about the structure of EPSs is becoming increasingly clear, details of the mechanisms of EPS action in various systems are only beginning to be understood. The multifarious properties of EPSs create a possibility of their wide future application in both biotechnology and medicine.

## Abbreviations

Arab: Arabinose
CTAB-PS: Isolation of EPS by cetyltrimethylammonium bromide precipitation
EPS: Exopolysaccharide
ET: Ethylene
FBBs: Fungal–bacterial biofilmed biofertilizers
FRBs: Fungal–rhizobial biofilmed biofertilizers
Fuc: Fucose
Gal: Galactose
Glc: Glucose
GlcA: Glucuronic acid
GPC: Gel permeation chromatography
HPAEC: High performance anion exchange chromatography
IEC: Ion exchange chromatography
IFN: Interferon
IL: Interleukin
ISR: Induced systemic resistance
JA: Jasmonic acid
KSM: Potassium solubilizing microorganisms
Mal: Maltose
Man: Mannose
ME: Malt extract
MG: Monosodium glutamate
MSS: Mineral salt solution
NK-cell: Natural killer cell
PDB: Potato dextrose broth
PE: Potato extract
PPAR: Peroxisome proliferator-activated receptor
PS: Polysaccharide
PSM: Phosphate solubilizing microorganisms
Rha: Rhamnose
SA: Salicylic acid
SAR: Systemic acquired resistance
SEC: Size exclusion chromatography
SEC/MALS: The combination of SEC with multi-angle light scattering analysis (MALS)
TCA: Trichloric acetic acid
TNF: Tumor necrosis factor
VMSM: Vogel minimum salt medium
Xyl: Xylose
YE: Yeast extract
